# Beyond the home: rethinking heritage language maintenance as a collective responsibility

**DOI:** 10.3389/fpsyg.2025.1616510

**Published:** 2025-06-10

**Authors:** Seyma Inan, Yvette Renee Harris

**Affiliations:** ^1^The Department of Psychology, Mercyhurst University, Erie, PA, United States; ^2^The Department of Psychology, Miami University, Oxford, OH, United States

**Keywords:** heritage language, immigrant families, language maintenance and preservation, bilingualism, family language policy (FLP)

## Introduction: heritage language maintenance requires more than family effort

Heritage language maintenance (HLM) is an important aspect of preserving cultural identity, fostering intergenerational communication, and promoting inclusive multicultural societies. In multilingual communities, particularly those shaped by immigration, HLM reflects the ongoing efforts of families and individuals to preserve their linguistic and cultural heritage in the face of dominant language ideologies (Fisherman, 2001; Halsted, 2015). While traditionally viewed as the responsibility of immigrant families, a growing body of literature challenges this notion by highlighting the need for broader systemic, institutional, and societal support (Inan et al., 2024). In this opinion paper, we argue for reframing HLM as a collective responsibility involving educational institutions, policymakers, communities, and digital foundations, rather than relying solely on the efforts of families.

Many immigrant parents reveal a strong desire to preserve their heritage languages, as they perceive language as an emotional and symbolic bridge to their cultural identity, values, and connection with their children (Ozkaynak, 2023; Bayram and Wright, 2016). These efforts are often grounded in Family Language Policy (FLP), which refers to both explicit and implicit language management practices within the home environment (King et al., 2008; Nenonen, 2024). FLP encompasses parental decisions on which languages are spoken, how consistently they are used, and the ideologies that shape these practices (Wilson, 2020). Despite its centrality to language maintenance, FLP alone cannot account for the various sociopolitical and economic pressures that influence families' language practices and beliefs.

## Rethinking the limits of family language policy

Numerous studies underscore the role of parents' positive attitudes and engagement in fostering successful HLM (Tran et al., 2022; Inan et al., 2024). However, maintaining consistent language policies at home remains challenging, with research showing that only a third of families have explicit rules about language use, and even fewer enforce them regularly (Tran et al., 2022). Additionally, macro-level societal attitudes toward minority languages, alongside conflicting language ideologies in schools and communities, often create barriers that diminish the effectiveness of FLP (Cui and Gao, 2024).

Children's bilingualism is not simply the product of exposure at home; it develops through dynamic, interactive experiences across multiple contexts. As Bialystok (2012) asserts, bilingual development is influenced by a complex interplay of individual, familial, and environmental factors. Thus, viewing HLM only through the lens of FLP neglects these broader dynamics. Effective language planning must consider the intersecting influences of institutional support, language ideologies, and public discourse (Kaplan and Baldauf, 2003).

Ginting (2020) and Inan et al. (2024) emphasize that while parental involvement is critical, it must be supported by external infrastructures that legitimize and facilitate heritage language use. Without this support, families often struggle to maintain language practices, especially when these efforts are not reinforced in educational or community settings. Consequently, we advocate for a more comprehensive, ecological approach to HLM that includes collaborative responsibility from multiple sectors. Those multiple sectors are discussed below.

## Educational institutions as pillars of heritage language maintenance

Schools serve as critical agents in shaping language attitudes, ideologies, and practices. Educational institutions can play a central role in reinforcing HLM through inclusive curricula, bilingual programs, and educator training. Unfortunately, many mainstream education systems in the United States and other monolingual-oriented countries fail to reflect the linguistic diversity of their student populations (Kunduz, 2022). This failure often leads to the marginalization of heritage languages and reinforces assimilationist pressures, which are not healthy for integration of immigrant families (Inan et al., 2024).

However, programs such as Dual Language Immersion (DLI) offer promising alternatives. These programs integrate students from diverse linguistic backgrounds and promote biliteracy, academic achievement, and cross-cultural understanding (Lindholm-Leary, 2023). Participation in bilingual programs has been shown to foster students' cultural identity and linguistic confidence while creating a more equitable learning environment (Garcia and Wei, 2023). Schools that adopt inclusive curricula can signal to students and families that their languages and cultures are valued.

Teachers, too, play a pivotal role in shaping how heritage languages are perceived and supported in the classroom. Carreira and Kagan (2018) advocate for culturally responsive pedagogy that equips educators to meet the diverse needs of heritage language learners. Comprehensive teacher training is essential in enabling educators to navigate linguistic diversity effectively and to engage with students' cultural contexts meaningfully (Kunduz, 2022). As Willoughby (2024) argues, inclusive curricula not only enrich the learning experience but also empower students to maintain connections with their linguistic roots.

Inan et al. (2024) further emphasize the transformative potential of schools as allies in HLM, particularly when educational practices are aligned with families' linguistic goals. However, to achieve this alignment, there must be systemic support at the policy level. Schools must be resourced with bilingual materials (e.g., welcoming signs in multiple world languages), trained staff, and administrative policies that reflect a commitment to multilingualism.

## Community networks and digital platforms: expanding access and support

Beyond the classroom, community organizations have long supported HLM by offering language classes, cultural events, and other enrichment opportunities (Park and Sarkar, 2008). These organizations serve as safe and affirming spaces where bilingual families can build networks, reinforce their language practices, and strengthen their sense of belonging. Such initiatives are particularly crucial in areas where formal educational support for heritage languages is lacking.

Kondo-Brown (2005) found that children who participate in community-based heritage language schools often demonstrate higher proficiency and stronger cultural identity. However, the reach and effectiveness of these programs are often limited by funding constraints, volunteer capacity, and the varying degrees of parental involvement. Community support structures also vary depending on the sociopolitical climate and local attitudes toward bilingualism (Sugiyanta, 2020).

In the digital age, online platforms have emerged as additional tools for supporting HLM. Digital resources such as language learning apps, bilingual storybooks, social media groups, and YouTube channels provide interactive and accessible ways for families to practice their heritage languages outside of formal contexts (Hu et al., 2014; Visonà and Plonsky, 2019). These tools are especially valuable for younger generations who are digitally literate and accustomed to virtual engagement.

Parental beliefs about the utility of bilingualism also shape how families navigate their HLM journeys. Some families view bilingualism as a socioeconomic asset that can enhance career prospects and global mobility, motivating them to seek out both community and digital resources (Inan et al., 2024). However, others—particularly those facing economic pressures—may prioritize dominant language acquisition for perceived practical advantages (Idaryani and Fidyati, 2023). Mulgrew et al. (2021) illustrate this tension in the context of Irish language speakers, where societal attitudes and employment concerns lead many parents to deprioritize heritage language use.

These examples demonstrate the need for coordinated community and digital initiatives that counteract language shift by promoting the long-term value of heritage languages and ensuring equitable access to resources across socioeconomic strata.

## Policy implications: addressing structural barriers

The barriers to HLM are not only personal or familial—they are structural. In the United States, language policies have historically prioritized English at the expense of minority languages, contributing to the destruction of linguistic diversity (Kunduz, 2022). Schools often lack the institutional frameworks necessary to support bilingual students, and families frequently navigate conflicting language ideologies with limited guidance.

To address these barriers, robust policy interventions are necessary. Government support for bilingual education, teacher training, and community programming can help legitimize and institutionalize heritage language instruction. Policies must explicitly value multilingualism as a public good and ensure equitable access to educational and cultural resources for all language communities (Potowski and Carreira, 2004).

International examples offer instructive models. In Canada, official bilingual policies have helped normalize the use of both English and French in education and public life (Adesope et al., 2010). In Australia, community-based language initiatives align with national goals of multiculturalism, demonstrating the potential of grassroots efforts supported by institutional backing (Taylor-Leech and Tualaulelei, 2021). These cases reveal that meaningful language maintenance is possible when policy, education, and community efforts are synchronized.

Nenonen (2024) reinforces the necessity of systemic support in navigating the complexities of language maintenance. Kupisch and Rothman (2016) echo this by emphasizing the importance of context-sensitive approaches that are informed by sociolinguistic realities. Ultimately, fostering sustainable bilingualism requires investment, collaboration, and commitment at multiple levels.

## A call for collective commitment

In conclusion, we argue that heritage language maintenance is a deeply social process that extends beyond the home. While families remain central to bilingual development, they cannot sustain these efforts in isolation. A holistic and sustainable approach to HLM requires coordinated involvement from educational institutions, policymakers, community organizations, and digital media platforms.

This opinion paper has underscored the limitations of placing the burden solely on immigrant families and has outlined a vision for shared responsibility. Schools must implement inclusive curricula and support bilingual educators; community organizations must be resourced to offer meaningful engagement opportunities; digital platforms must be accessible and culturally relevant; and policymakers must enact legislation that affirms linguistic diversity as a societal value.

Future research should explore how digital technologies intersect with socioeconomic status to shape language practices, and how schools can better align with the language goals of diverse families. Recognizing the collective dimensions of HLM not only supports bilingual children but also strengthens cultural diversity, economy, and contributes to a more equitable society.

By embracing this collective framework, multilingual societies can ensure that heritage languages are not only preserved but celebrated as important components of our shared human experience.
